# Fabrication and characterization of branched carbon nanostructures

**DOI:** 10.3762/bjnano.7.116

**Published:** 2016-09-05

**Authors:** Sharali Malik, Yoshihiro Nemoto, Hongxuan Guo, Katsuhiko Ariga, Jonathan P Hill

**Affiliations:** 1 Institute of Nanotechnology, Karlsruhe Institute of Technology (KIT), D-76131 Karlsruhe, Germany; 2 WPI-Center for Materials Nanoarchitectonics, National Institute for Materials Science (NIMS), Namiki 1-1, Tsukuba, Japan

**Keywords:** branched multiwalled carbon nanotubes, carbon nanostructures, carbon nanotubes, graphene nanoribbons, multiwalled carbon nanotubes

## Abstract

Carbon nanotubes (CNTs) have atomically smooth surfaces and tend not to form covalent bonds with composite matrix materials. Thus, it is the magnitude of the CNT/fiber interfacial strength that limits the amount of nanomechanical interlocking when using conventional CNTs to improve the structural behavior of composite materials through reinforcement. This arises from two well-known, long standing problems in this research field: (a) inhomogeneous dispersion of the filler, which can lead to aggregation and (b) insufficient reinforcement arising from bonding interactions between the filler and the matrix. These dispersion and reinforcement issues could be addressed by using branched multiwalled carbon nanotubes (b-MWCNTs) as it is known that branched fibers can greatly enhance interfacial bonding and dispersability. Therefore, the use of b-MWCNTs would lead to improved mechanical performance and, in the case of conductive composites, improved electrical performance if the CNT filler was better dispersed and connected. This will provide major benefits to the existing commercial application of CNT-reinforced composites in electrostatic discharge materials (ESD): There would be also potential usage for energy conversion, e.g., in supercapacitors, solar cells and Li-ion batteries. However, the limited availability of b-MWCNTs has, to date, restricted their use in such technological applications. Herein, we report an inexpensive and simple method to fabricate large amounts of branched-MWCNTs, which opens the door to a multitude of possible applications.

## Introduction

Lighter, stronger materials such as nanocarbon composites offer benefits for applications such as transport, energy storage/conversion and bone/tooth replacement. Hence, the mechanical properties of CNTs are utilized in reinforcing polymer composites [1–4], and their electrical conductivity is utilized for conducting polymers [4–6]. Under tensile load only the outermost layers of MWCNTs are involved as the relatively weak (van der Waals) bonding between the outer layers and the inner layers leads to slippage – the so called “sword in sheath” failure mechanism, which reduces the load-bearing capacity [1,7]. However, under compressive load this slippage leads to the very useful elastic deformation of MWCNTs [8–9].

For all nanoscale reinforcing component materials (NRCMs) including nanocarbons such as graphene, there remain two well-known, long standing issues which are widely recognized as being critical for the development of mechanically efficient nanocomposites: a) inhomogeneous dispersion of the filler [10] and (b) insufficient strength of the interphase between the filler and the matrix [11]. However, these issues can be addressed by utilizing branched multiwalled carbon nanotubes (b-MWCNTs). It is known from theory and simulation experiments [12–14] that branched fibers can greatly enhance interfacial bonding and dispersability. Such an approach is exemplified by the process of adding straw (branched plant fibers) to mud to make stronger bricks which has been used since the Neolithic period, i.e., before 3400 BC [15]. More recently, Masselter et al. have correlated the functional morphology of branching in plants with mechanical behavior and concluded that the concepts generated have a high potential for implementation in the development of branched fiber-reinforced technical composites [16]. With respect to electrical and electronic properties, it is well known that in carbon nanotube networks the junction resistance controls the overall performance [17]. Therefore, in addition to b-MWCNTs/composite applications, the enhanced electrical properties of networks arising within this new material has major potential benefits for design, development and production of supercapacitors, solar cells and Li-ion batteries.

The first experimental observation of branched carbon nanotubes appears to have been in 1995, when after using an arc-discharge method L-, Y- and T-shaped MWCNTs were produced [18]. Subsequently, branched CNTs have been fabricated using a variety of methods, which include pyrolysis of metallocenes [19–20], nanowelding [21], catalytic CVD [22–23], carbon infiltration of MWCNTs [24], templating [25] and chemical functionalization [26]. However, none of these methods are easily industrially scalable. Herein, we report a cheap and simple method to fabricate large amounts of branched MWCNTs in order to address the well-known problems of adequate dispersion and sufficiently strong interfacial bonding required for large-scale applications.

**Figure 1:**
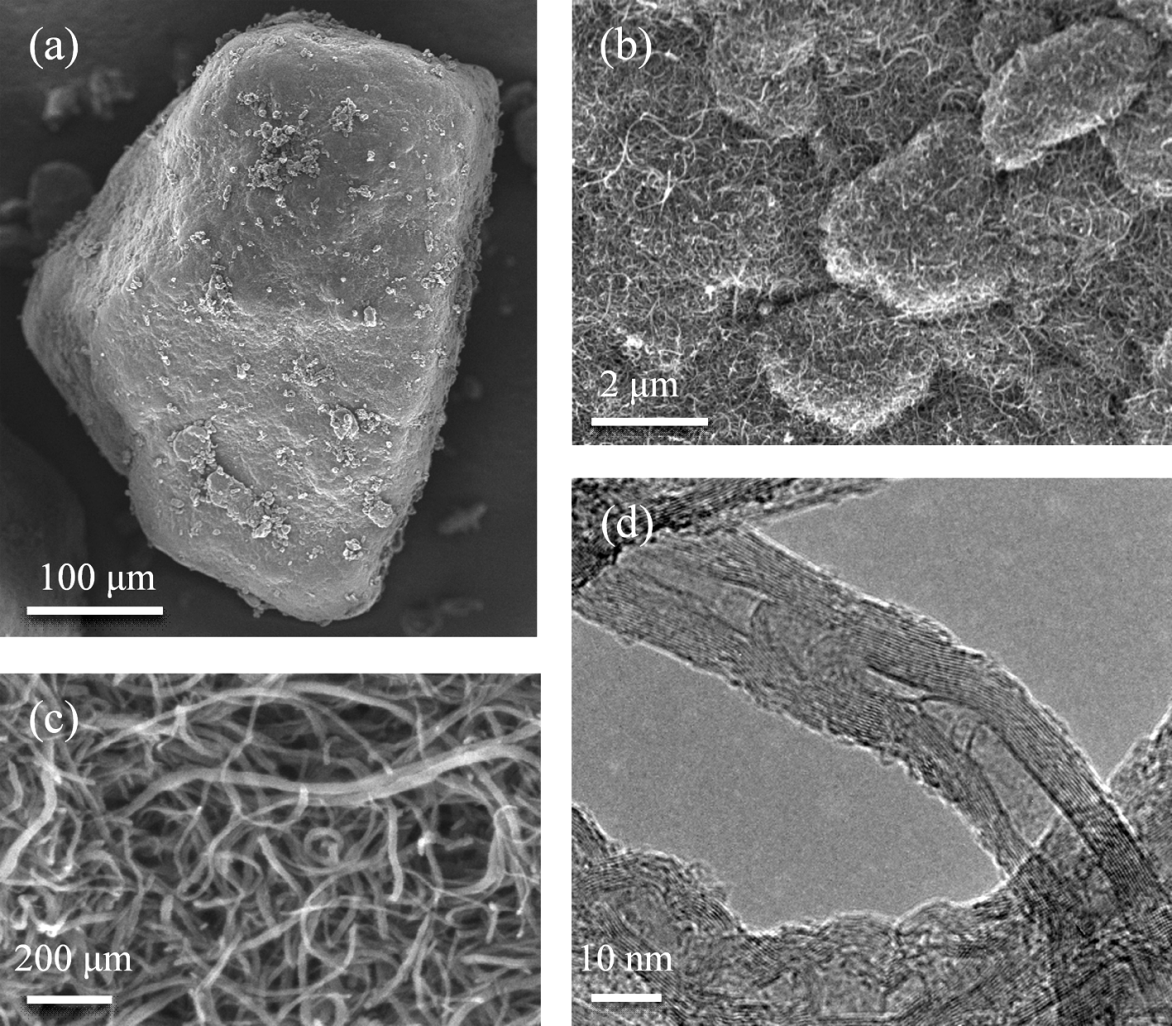
a) SEM overview of a Baytubes agglomerated pellet; b, c) SEM details of the MWCNTs; d) TEM detail of typical MWCNTs.

As a starting material it was decided to use the commercially available MWCNTs, namely Baytubes^©^. Other workers have shown using high resolution transmission electron microscopy (HRTEM) that Baytubes^©^ are parallel-walled in long closed sections and that they are strong and relatively pure [27]. They form around a catalyst and are supplied as loosely agglomerated pellets (Figure 1), which has advantages regarding health, safety and environment. According to the data sheet Baytubes^©^ belong to the category of short, thin tangled MWCNTs for which no “fibre-like” pathogenic behaviour is expected based on the available data. The MWCNTs used in this research were Baytubes^©^ C 150P (Bayer Material Science A.G., Leverkusen, Germany).

## Results and Discussion

**Figure 2:**
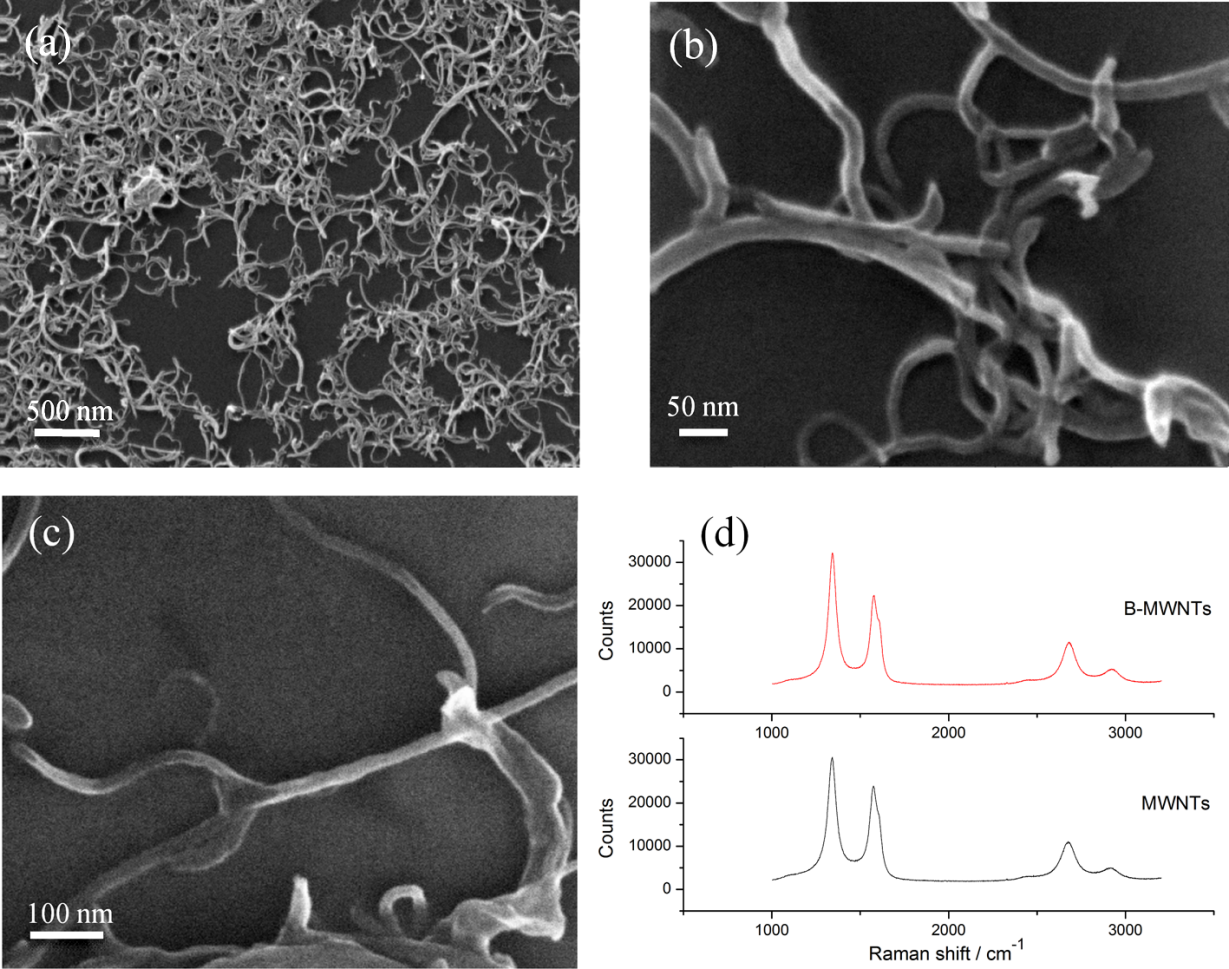
a) Helium ion microscope (HeIM) overview of b-MWCNTs and b) HeIM detail of b-MWCNTs; c) SEM detail of unzipped and branched-MWCNTs; d) Raman spectra of as received MWCNTs (bottom spectrum) and b-MWCNTs (top spectrum) – both at 532 nm.

The idea of this work is to “unzip” the MWCNTs and to allow them to re-roll and form branched MWCNTs. This works for MWCNTs like Baytubes^©^, because they have parallel sides [27] and so they can unzip to form layered nanoribbons. Since they are in long closed sections (Figure 1d) we can assume that the end of these sections acts like a “rip-stop” so that the unzipped tubes can re-roll from these points to form branched structures (Figure 2a–c). The Raman spectra of the as-received MWCNTs (bottom spectrum) and of the branched-MWCNTs (top spectrum) are shown in (Figure 2d). Both sets of spectra are typical of MWCNTs and the *I*_D_/*I*_G_ values [28–29] of the as-received MWCNTs and the b-MWCNTs are 1.26 and 1.43, respectively. This indicates that they both have very similar defect densities.

**Figure 3:**
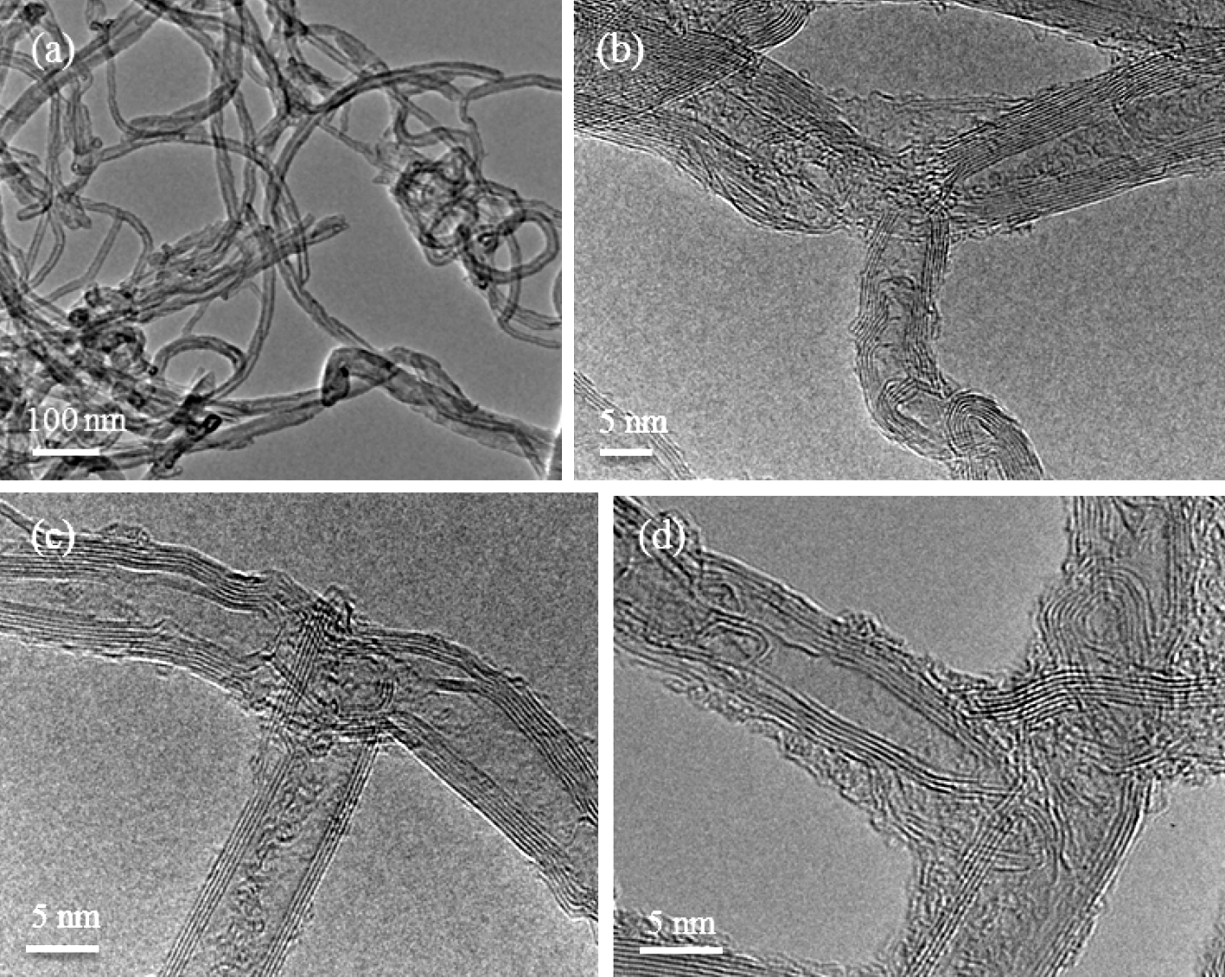
a) HRTEM overview of branched-MWCNTs and b) HRTEM detail of Y-pattern b-MWCNTs; c, d) HRTEM detail of T-pattern b-MWCNTs.

**Figure 4:**
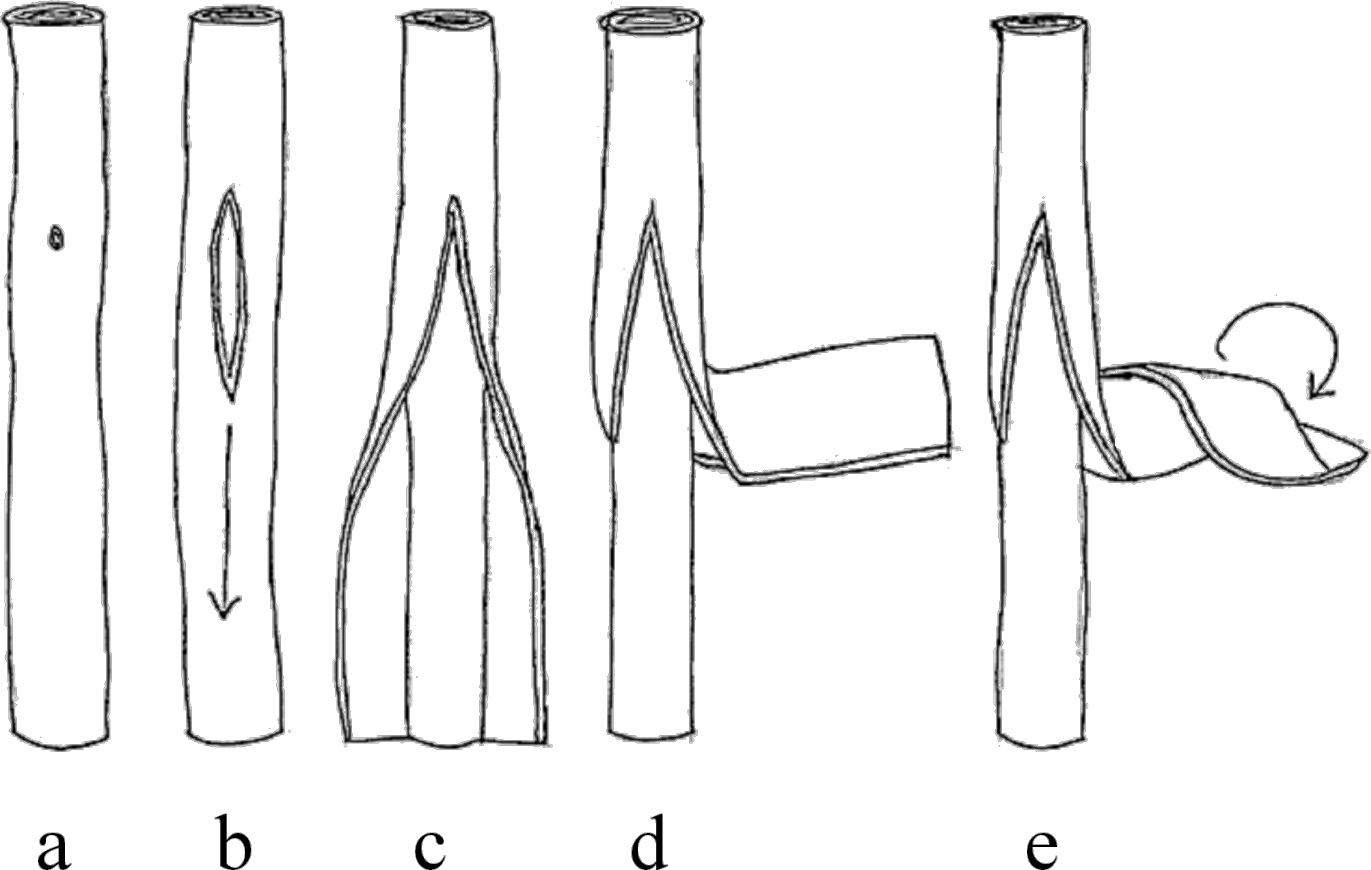
A schematic diagram of the suggested “unzipping” and “re-rolling” sequence: a) formation of unzipping point; b) onset of unzipping; c) unzipping and onset of peeling of inner parallel tubes; d) outer layers peeling out as a sheet; e) onset of re-rolling of outer layers.

The mechanism of unzipping MWCNTs to form graphene nanoribbons is well known from research by Hirsch [30] and Dai et al. [31] but the procedure is complex and the yield is low [32–34]. However, as we show here, if the aim is to make branched-MWCNT then the procedure is much simpler. Thus, as-received MWCNTs were heated to 500 °C, which results in the introduction of defects that later act as the “unzipping” points. The procedure has the additional benefit of cleaning the tubes as substantiated from the Raman (Figure 2d) and HRTEM data (Figure 3) confirming the absence of carbon impurities or residual catalyst material. The heated MWCNTs were then sonicated in ethanol which causes the MWCNTs to unzip and re-roll. Figure 4 shows a suggested schematic sequence in agreement with observations by Kaner et al. [35] and Geim [36] indicating that nanoribbons tend to re-roll unless prevented from doing so.

**Figure 5:**
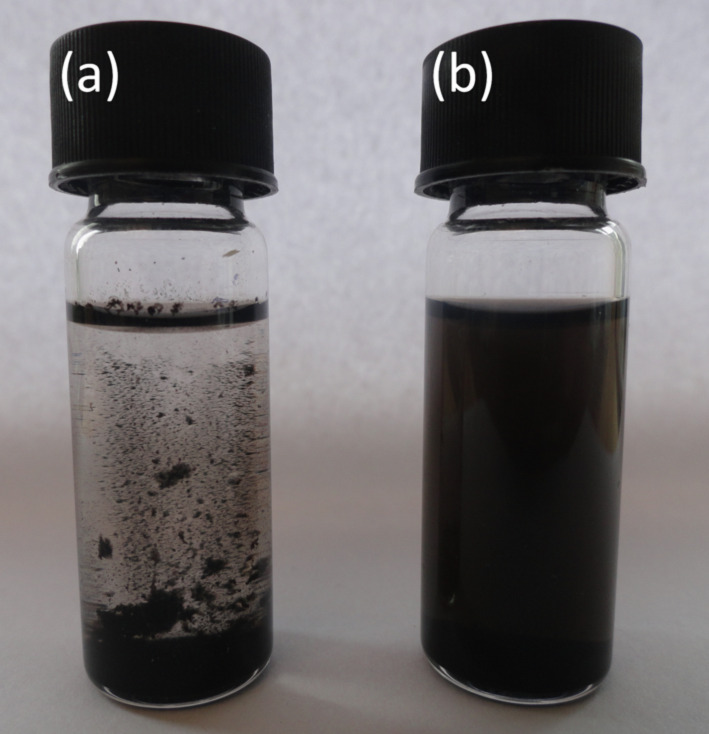
(a) Dispersion of MWCNTs starting material; (b) dispersion of b-MWCNTs.

Dispersions of the starting MWCNTs and b-MWCNTs were compared by dispersing 1 mg of each material in 4 mL of ethanol. These dispersions were then centrifuged at 3500*g* for 2 h and then left to stand for 24 h. The liquid suspensions are shown in Figure 5. The b-MWCNTs clearly show better dispersability compared to the starting MWCNTs.

As no surfactants or expensive polymers are needed for this process, it can be described as inexpensive and easy and it results in clean b-MWCNTs (Figure 3). The yield is estimated (using the methodology by Dai et al. [31]) to be about 60% branched MWCNTs. The Y-branched MWCNTs fabricated here (Figure 3b) obey geometric conservation laws that are consistent with earlier theory [37] in that the angle between two neighboring branches should be 120°. Therefore, in this instance theory and experiment are in agreement, even though this agreement is not “perfect” as in practice defects will certainly be present.

The unzipping and re-rolling process appears to reduce the number of walls on the MWCNTs compared to those for the as-received tubes (Figure 1d) and it is also possible that some of the tubes re-roll to form carbon nano-scrolls (CNS) [35,38–40]. This possibility is evidenced in Figure 3c where the number of walls of the three parts of the “T” pattern does not meet the “Russian Doll” MWCNTs requirement of *N* (left) = *N* (right) + *N* (down) or *N* (right) = *N* (left) + *N* (down). The Raman spectra show that the b-MWCNTs have a similar defect density as the starting material, which shows that the material has not been damaged by the fabrication procedure. The EDX analysis of the MWCNTs starting material is 100% carbon (the data sheet indicates a carbon purity of over 95%). The EDX analysis of the branched-MWNTs is 96% carbon and 4% oxygen. This is consistent with the initial heating in air, which removes amorphous impurities and etches/oxidizes the MWCNTs at defect sites without damaging the sidewalls [31]. In many nanocarbon material applications the presence of residual surfactant or organic residues can be a problem, but this is not the case here.

**Figure 6:**
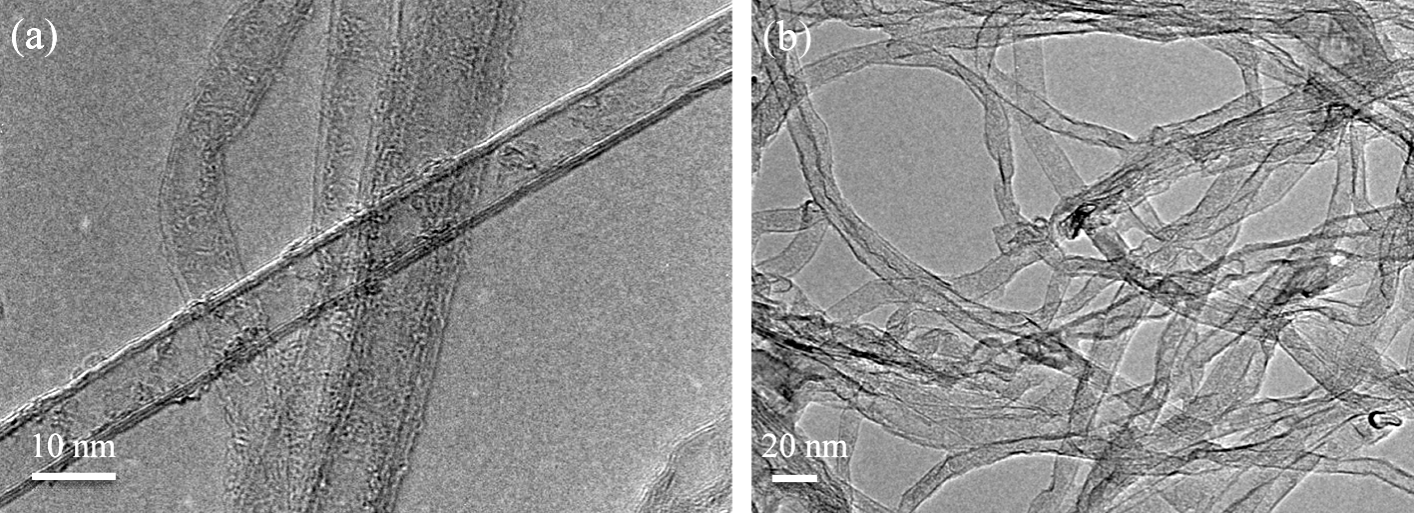
TEM overview of a) thin MWCNTs starting material and b) graphene nanoribbons after treatment.

The experimental procedure produces b-MWCNTs when using thick MWCNTs (more than a few walls). However, when the same procedure is used with thin MWCNTs (e.g., triple-walled MWCNTs, Figure 6a), synthesised by a “water-assisted” CVD method [41], graphene nanoribbons are produced in small yields (Figure 6b) which is consistent with earlier works by Hirsch [30] and Dai et al. [31].

## Conclusion

In summary, we have described herein a new, simple and cheap route to fabricate large amounts of branched MWCNTs using widely available commercial MWCNTs. We have demonstrated a facile procedure for making clean branched nanostructures and this opens promising avenues for the development and manufacturing of nanocarbon composites for a variety of commercial applications. The fabrication and testing of composite materials with branched MWCNTs as well as measurements of electrical conductivity are currently in progress.

## Experimental

The MWCNTs used in this research were Baytubes^©^ C 150P (Lot-No E0009AA007, Drum-No 033), supplied by Bayer Material Science A.G. (Leverkusen, Germany).

### Preparation of b-MWCNTs

Commercial MWCNTs were heated in air to 500 °C for 2 h to give the “stage I”-modified MWCNTs. Then a small amount of these were sonicated in ethanol (30 mL) for 8 h (ultrasonic denerator, model GSCVP 150 at ca. 80% power). The dispersion was centrifuged at 3500*g* for 90 min to give the “stage II”-modified MWCNTs, which are the b-MWCNTs. The supernatant was spotted onto lacey carbon Cu TEM grids and onto polished Si chips for subsequent characterization.

### Preparation of graphene nanoribbons

A “water-assisted” CVD process as reported earlier [41–42] was used to fabricate thin walled MWCNTs (ca. three walls), which were subsequently heated in air to 500 °C for 2 h. Subsequently, ca. 10 mg of this material was sonicated (UP200s Dr. Hielscher, 200 W, 24 Hz, 0.5 cycles, 60% amplitude) in ethanol (4 mL) for 1 h. The resultant dispersion was spotted onto plain Cu TEM grids and polished Si chips for subsequent characterization.

### Characterization

Quality and morphology of the fabricated branched nanostructures were scrutinised by Raman spectroscopy (Renishaw inVia), TEM (Tecnai F20 ST at 200 kV), HRTEM (Jeol ARM at 120 kV), SEM (Zeiss Ultra-Plus at 5 kV), SEM (Zeiss Leo 1530 at 10 kV with Oxford X-Max 50 EDX ) and helium ion microscopy (HeIM, Zeiss Orion at 30 kV).
